# Fear of Cancer Recurrence and Associated Factors in Lymphoma Survivors and Their Family Caregivers: A Cross‐Sectional Study

**DOI:** 10.1002/cam4.70561

**Published:** 2025-03-29

**Authors:** Taha Koray Sahin, Ezgi Aysu Sahin, Hande Nur Gungor, Deniz Can Guven, Ibrahim Barista, Serkan Akin

**Affiliations:** ^1^ Department of Medical Oncology Hacettepe University Cancer Institute Ankara Turkey; ^2^ Department of Internal Medicine, Faculty of Medicine Hacettepe University Ankara Turkey; ^3^ Medical Oncology Clinic Health Sciences University, Elazig City Hospital Elazig Turkey

**Keywords:** anxiety, cancer, caregiver, fear of cancer recurrence, lymphoma, oncology, quality of life

## Abstract

**Background:**

Fear of cancer recurrence (FCR) is a pervasive concern among lymphoma survivors and their family caregivers, influencing psychological and physical health. Given the substantial burden of FCR, identifying its predictors is crucial for targeted interventions that could enhance palliative care. We aimed to evaluate the prevalence of FCR in lymphoma survivors and their caregivers, as well as associated factors

**Methods:**

A total of 118 patients with lymphoma, along with their family caregivers, were recruited from Hacettepe University Cancer Institute between March 2024 and May 2024. Psychological assessments were conducted using the Depression Anxiety Stress Scales (DASS‐21), the Fear of Cancer Recurrence Inventory‐Short Form (FCRI‐SF) and the Functional Assessment of Cancer Therapy‐Lymphoma (FACT‐Lym)

**Results:**

High levels of FCR were experienced by 50.8% (*n* = 60) of lymphoma survivors and 57.6% (*n* = 68) of their caregivers. There was a positive correlation between the FCR of the survivors and caregivers (*r* = 0.349, *p* < 0.001). Poor overall quality of life (QoL) (aOR: 4.279, 95% CI: 1.738–10.531, *p* = 0.002), recent diagnosis (< 3 year) (aOR: 5.135, 95% CI: 1.852–14.238, *p* = 0.002), survivors' anxiety (aOR: 2.540, 95% CI: 1.014–6.363, *p* = 0.002) and caregivers' FCR (aOR: 2.970, 95% CI: 1.119–7.879, *p* = 0.029) were associated with high levels of FCR in lymphoma survivors.

**Conclusion:**

We observed high FCR levels in over half the survivors with lymphoma and a higher FCR risk in patients with anxiety, poor QoL and caregiver FCR. These findings highlight the critical need for developing comprehensive care plans and interventions targeting FCR in patients with lymphoma.

## 1 Introduction

Lymphomas represent a heterogeneous group of malignant lymphoid neoplasms with significantly different prognosis and treatment and are broadly categorized into two groups: Hodgkin lymphoma (HL) and non‐Hodgkin lymphoma (NHL) [1]. With the advent of novel treatment options, the 5‐year relative survival rate has reached over 90% for HL and about 80% for NHL. However, approximately 30%–40% of patients with lymphoma eventually experience recurrence [2, 3]. Given the high survival rates and young age at diagnosis, lymphoma survivors are susceptible to several treatment‐induced late effects, such as the development of secondary malignancies and cardiotoxicity [4]. Additionally, they also confront high levels of anxiety, depression, and fear of recurrence (FCR), highlighting the need for comprehensive psychosocial assessment during survivorship [5, 6, 7].

Fear of cancer recurrence (FCR) has been defined as “fear, worry, or concern that cancer will come back or progress” [8]. FCR is a significant unmet need among cancer survivors. Recent studies report prevalence rates around 50% when using validated tools like the Fear of Cancer Recurrence Inventory‐Short Form (FCRI‐SF), depending on the cutoff score applied [9, 10]. A surge in FCR research has yielded significant progress, yet knowledge gaps persist regarding prevalence across different tumor types, underlying biological pathways, and optimal interventions [10, 11]. Patients with lymphoma, in particular, are markedly underrepresented in FCR studies, despite unique disease‐specific and demographic factors that may influence FCR. Furthermore, while lymphomas often have high survival rates—such as the 5‐year survival rate of 87%–96% for HL and the potential for late relapses and the need for long‐term monitoring may sustain persistent FCR [12]. Unique relapse patterns, like Richter transformation in chronic lymphocytic leukemia or aggressive shifts in follicular lymphoma, add to this burden [13]. Despite growing recognition of these challenges, limited data exist regarding associated factors of FCR in lymphoma survivors.

Whilst caregivers (partners, family members, or friends) exhibit similar or even higher levels of FCR when compared to survivors, the existing literature on caregiver FCR, though emerging, remains limited, particularly in terms of research focused on specific caregiver dynamics in lymphoma populations [14, 15]. Similarly to survivors, FCR in caregivers is persistent and associated with impaired quality of life (QOL), lower functioning, and higher emotional symptoms (e.g., anxiety) [16, 17]. Building upon the family model by Mellon et al. [18], the FCR experienced by survivors and caregivers influences each other, resulting in an interdependent dynamic between their responses to cancer. Therefore, our study aimed to assess the prevalence of FCR in lymphoma survivors and their caregivers, as well as to identify its predictive factors.

## 2 Materials and Methods

### 2.1 Design and Participants

A cross‐sectional survey was conducted at Hacettepe University Cancer Institute from March to May 2024. We included lymphoma survivors who had completed any cancer treatment at least 3 months ago and were receiving regular follow‐up care, along with their caregivers in pairs. Patients' inclusion criteria were as follows: (1) having completed lymphoma treatment; (2) the presence of a caregiver providing care for a family member, partner, or friend; (3) 18 years of age or older; and (4) agreement to sign the written informed consent and completion of all required questionnaires. We excluded participants based on the following criteria: (1) history of pre‐existing neurocognitive impairment; (2) inability to complete questionnaires due to communication issues; and (3) caregivers' unavailability to participate.

During the recruitment period, all eligible lymphoma survivors attending follow‐up visits at our outpatient clinic were approached for potential participation. Following routine medical examinations, participants were informed about the study's purpose and details. Both survivors and their caregivers were then invited to participate and complete surveys if they agreed.

### 2.2 Measurements

#### 2.2.1 Study Participants

Baseline demographic and clinical data of participants were collected via self‐reported questionnaires and retrospective chart review of the electronic health record. The collected data included current age, gender, employment status, marital status, education level, family income, medical co‐morbidities, previous anti‐cancer treatments (chemotherapy, radiotherapy, immunotherapy, and stem cell transplantation), and time since diagnosis of lymphoma (based on median value).

#### 2.2.2 Fear of Cancer Recurrence Inventory‐Short Form (FCRI‐SF)

The FCRI‐SF was used to measure the severity of FCR using 9 items rated on a five‐point Likert scale from 0 (not at all) to 4 (all the time) [19]. One item (#5) is reverse‐scored before items are summed to create a total score. Total scores range from 0 to 36, and higher scores indicate higher FCR. In this study, an FCRI‐SF score of 13 or higher was considered indicative of higher FCR, consistent with previous studies [20]. The FCRI‐SF has been previously validated in Turkey [21], and widely employed to examine FCR in Turkish cancer survivors. The internal consistency of the FCRI‐SF in our study was high (Cronbach's alpha = 0.85).

#### 2.2.3 Depression, Anxiety, and Stress Scale (DASS‐21)

The Depression Anxiety Stress Scale‐21 (DASS‐21) is a self‐report screening tool derived from the longer 42‐item form [22]. The scale was used to measure depression, anxiety, and stress symptoms in both survivors and caregivers. Each factor consists of seven items. Participants were asked to rate each statement using four‐point scale ranging from 0 (did not apply to me at all) to 3 (applied to me very much) with higher scores indicating higher levels of depression, anxiety, and stress. Established cut‐off scores on the DASS‐21 categorize the severity of symptoms for each subscale [22]. The Turkish version was validated by Yılmaz et al. [23]. In this study, Cronbach's alpha values were high: 0.860 for depression, 0.732 for anxiety, and 0.804 for stress.

#### 2.2.4 QOL

QOL was measured using the Functional Assessment of Cancer Therapy‐Lymphoma (FACT‐Lym) scale [24]. The Turkish version of FACT‐Lym Scale was provided by the Functional Assessment of Chronic Illness Therapy (FACIT) organization and was validated by Sezgin et al. [25]. The FACT‐Lym comprises the FACT‐General (27 items) and the lymphoma‐specific subscale (15 items). The FACT‐General questionnaire covers four subscales: social, physical, emotional, and functional well‐being, while the lymphoma‐specific subscale addresses 15 additional lymphoma‐related items and long‐term effects of treatment to assess their influence on health‐related QOL. High scores indicate better QOL. The internal consistency of the FACT‐Lym in this study had a Cronbach's alpha value of 0.795.

### 2.3 Statistical Analysis

All statistical analyses were performed with SPSS software (version 25.0, IBM, Armonk, NY). Scores for FCRI‐SF, DASS‐21, and FACT‐Lym were calculated following their respective manuals. Continuous variables were expressed as median and interquartile range (IQR), while categorical variables were summarized as percentages and frequencies. We used descriptive statistics to summarize these characteristics and *χ* ^2^ tests for categorical variables. Continuous variables were compared using independent samples *t*‐test for normally distributed data, and the nonparametric Mann–Whitney *U*‐test was employed for non‐normally distributed data. Pearson correlation analysis was used to explore the correlation between lymphoma survivors' FCR and depression, anxiety, stress and their caregivers' FCR. Subsequently, bivariate logistic regression analysis was performed, and variables with a *p*‐value < 0.10 were selected for inclusion in the multivariable logistic regression model. To identify independent variables associated with a high FCR score (≥ 13), a multivariable logistic regression model was applied using a backward stepwise selection approach. Odds ratios (OR) with 95% confidence intervals (CIs) were reported for the factors associated with FCR levels.

## 3 Results

A total of 160 patients applied to Hacettepe University Cancer Institute between March and May 2024. Of these, 42 patients were excluded based on predefined criteria, including four patients with a history of pre‐existing neurocognitive impairment, four patients unable to complete questionnaires due to communication issues, and 34 patients whose caregivers were unavailable to participate. Ultimately, 118 patient‐caregiver dyads met the inclusion criteria and were included in the study. Over half the survivors were male (53.4%), had a high‐school degree or lower education (50.2%), and were employed (61%). The median age of the survivors was 51 (IQR: 38–59). Most of the survivors (61.9%) had NHL and no recurrence of disease (78.8%). Treatment details reveal that 81.4% of participants received chemotherapy, 32.2% received radiotherapy, 7.6% received immunotherapy, and 11% underwent hematopoietic stem cell transplantation (HSCT). Table 1 summarizes the participant characteristics in more detail. There were no significant differences between the FCR low and FCR‐high groups in age, sex, marital status, education, employment status, type of lymphoma, treatment. However, the lymphoma survivors who had high FCR were more likely to have a recent diagnosis of lymphoma (< 3 year) (*p* = 0.003), prior disease recurrence (*p* = 0.017), high anxiety score (*p* = 0.006), low FACT‐Lym total score (*p* < 0.001).

**TABLE 1:** Patients characteristics and their relationship with fear of cancer recurrence (*n* = 118).

		Total (*n* = 118)	Fear of cancer recurrence		*p*
			Low (*n* = 58)	High (*n* = 60)	
Median age (years), IQR		51 (38–59)	51.5 (45–60)	49.5 (34–58)	0.588
Sex	Male	63 (53.4)	34 (58.6)	29 (48.3)	0.263
	Female	55 (46.6)	24 (41.4)	31 (51.7)	
Education	High school or below	65 (55.1)	35 (60.3)	30 (50)	0.259
	University or above	53 (44.9)	23 (39.7)	30 (50)	
Marital status	Single or divorced	33 (28)	15 (25.9)	18 (30)	0.617
	Married	85 (72)	43 (74.1)	42 (70)	
Employment	Employed	72 (61)	33 (56.9)	39 (65.0)	0.727
	Unemployed	46 (39)	25 (43.9)	21 (35.0)	
Family monthly income	< 30.000 TL	68 (57.6)	31 (53.4)	37 (61.7)	0.366
	> 30.000 TL	50 (42.4)	27 (46.6)	23 (38.3)	
Clinical variables					
Type of lymphoma	HL	45 (38.1)	25 (43.1)	20 (33.3)	0.275
	NHL	73 (61.9)	33 (56.9)	40 (66.7)	
Time since diagnosis	< 3 year	73 (61.9)	28 (48.3)	45 (75)	0.003
	≥ 3 year	45 (38.1)	30 (51.7)	15 (25)	
Recurrence	Yes	25 (21.2)	7 (12.1)	18 (30)	0.017
	No	93 (78.8)	51 (87.9)	42 (70)	
Treatment	Chemotherapy	96 (81.4)	46 (79.3)	50 (83.3)	0.575
	Radiotherapy	38 (32.2)	22 (37.9)	16 (26.7)	0.190
	Immunotherapy	9 (7.6)	2 (3.4)	7 (11.7)	0.093
	HSCT	13 (11.0)	7 (12.1)	6 (10)	0.720
Psychological variables					
DASS‐21					
Depression	Yes	38 (32.2)	14 (24.1)	24 (40)	0.065
	No	80 (67.8)	44 (75.9)	36 (60)	
Anxiety	Yes	62 (52.5)	23 (39.7)	39 (65)	0.006
	No	56 (47.5)	35 (60.3)	21 (35)	
Stress	Yes	24 (20.3)	8 (13.8)	16 (26.7)	0.082
	No	94 (79.7)	50 (86.2)	44 (73.3)	
FACT‐Lym					
Physical well‐being	≥ Median	70 (59.3)	44 (75.9)	26 (43.3)	< 0.001
	< Median	48 (40.7)	14 (24.1)	34 (56.7)	
Social well‐being	≥ Median	62 (52.5)	32 (55.2)	30 (50)	0.574
	< Median	56 (47.5)	26 (44.8)	30 (50)	
Emotion well‐being	≥ Median	56 (47.5)	35 (60.3)	21 (35)	0.006
	< Median	62 (52.5)	23 (39.7)	39 (65)	
Functional well‐being	≥ Median	66 (55.9)	28 (48.3)	38 (63.3)	0.100
	< Median	52 (44.1)	30 (51.7)	22 (36.7)	
Lymphoma subscale	≥ Median	64 (54.2)	42 (72.4)	22 (36.7)	< 0.001
	< Median	54 (45.8)	16 (27.6)	38 (63.3)	
FACT‐Lym (total)	≥ Median	62 (52.5)	43 (74.1)	19 (31.7)	< 0.001
	< Median	56 (47.5)	15 (25.9)	41 (68.3)	

Abbreviations: DASS‐21, the depression, anxiety and stress scale—21 items; FACT‐Lym, the functional assessment of cancer therapy—lymphoma; HL, Hodgkin lymphoma; HSCT, hematopoietic stem cell transplantation; NHL, non‐Hodgkin lymphoma.

The median age of caregivers was 48 years (IQR 35–56). Caregivers were mostly female (55.9%), and the majority were spouses/partners (57.6%), followed by children (18.6%), parents (12.7%), and others, including siblings, friends, or professional caregivers (11.0%). Caregiver characteristics are shown in Table 2. Demographics and physchological variables were largely similar between the FCR low and FCR‐high groups, except for a significantly higher proportion of caregivers with anxiety (*p* = 0.001) and depression (*p* = 0.001) in the FCR‐high group.

**TABLE 2:** Caregivers characteristics and their relationship with fear of cancer recurrence (*n* = 118).

		Total (*n* = 118)	Fear of cancer recurrence		*p*
			Low (*n* = 50)	High (*n* = 68)	
Median age, years		48 (37–56)	49 (37–56)	48 (37–55)	0.872
Sex	Male	52 (44.1)	26 (52)	26 (38.2)	0.137
	Female	66 (55.9)	24 (48)	42 (61.8)	
Relationship	Spouse/partner	68 (57.6)	28 (56)	40 (58.8)	0.969
	Parent	15 (12.7)	7 (14)	8 (11.8)	
	Child	22 (18.6)	9 (18)	13 (19.1)	
	Other	13 (11.0)	6 (12)	7 (10.3)	
Education	High school or below	52 (44.1)	16 (32)	36 (52.9)	0.024
	University or above	66 (55.9)	34 (68)	32 (47.1)	
Marital status	Single or Divorced	23 (19.5)	10 (20)	13 (19.1)	0.905
	Married	95 (80.5)	40 (80)	55 (80.9)	
Family monthly income	< 30.000 TL	68 (57.6)	29 (58)	39 (57.4)	0.944
	> 30.000 TL	50 (42.4)	21 (42)	29 (42.6)	
Psychological variables					
DASS‐21					
Depression	Yes	47 (39.8)	11 (22)	36 (52.9)	0.001
	No	71 (60.2)	39 (78)	32 (47.1)	
Anxiety	Yes	65 (55.1)	19 (38)	46 (67.6)	0.001
	No	53 (44.9)	31 (62)	22 (32.4)	
Stress	Yes	33 (28)	11 (22)	22 (32.4)	0.216
	No	85 (72)	39 (78)	46 (67.6)	

Table 3 displays the FCR, depression, anxiety, and stress levels of patient‐caregiver dyads. The incidence of FCR was 57.6% in caregivers and 50.8% in survivors. The FCR level in caregivers was significantly higher than in patients (*p* = 0.037). The depression, anxiety, and stress levels of caregivers and the survivors were similar (*p* = 0.794 for depression, *p* = 0.435 for anxiety, and *p* = 0.149 for stress).

**TABLE 3:** Comparison of FCR, stress, anxiety, and depression among lymphoma patients and their caregivers.

Variables	Participants	Significant case^a^	Mean (SD)	*p*
FCR	Patients	60 (50.8)	13.11 (7.1)	0.037
	Caregivers	68 (57.6)	15.5 (8.4)	
Depression	Patients	38 (32.2)	3.7 (3.4)	0.794
	Caregivers	47 (39.8)	3.9 (3.5)	
Anxiety	Patients	62 (52.5)	3.7 (2.6)	0.435
	Caregivers	65 (55.1)	4.1 (4)	
Stress	Patients	26 (22)	4.9 (3.5)	0.149
	Caregivers	33 (28)	5.3 (3.7)	

Abbreviations: FCR, fear of recurrence; SD, standard deviation.

^a^ Significant case means score more than or equal to 13 in FCRI‐SF for FCR and score of 5,4,8 in DASS‐21 for depression, anxiety, and stress, respectively. This table compares psychological variables (FCR, depression, anxiety, and stress) between lymphoma survivors and their caregivers, using paired data for direct comparisons.

The correlations between FCR, depression, anxiety, and stress levels in patients and their caregivers are presented in Table 4. The findings indicated significant correlations for most paired psychological variables between patients and caregivers. Specifically, there was a significant correlation between patients' and caregivers' FCR (*r* = 0.349, *p* < 0.001), depression (*r* = 0.297, *p* < 0.05), and stress levels (*r* = 0.258, *p* < 0.05). Anxiety in patients showed a weaker positive correlation with caregivers' anxiety (*r* = 0.054, *p* = 0.676). Overall, significant positive correlations were observed for patients' FCR, depression, and stress with their caregivers' corresponding scores.

**TABLE 4:** Correlation analysis of paired psychological variables between lymphoma patients and their caregivers.

Variables	FCR^b^	Depression^b^	Anxiety^b^	Stress^b^
FCR^a^	0.349**	0.289*	0.110	0.258*
Depression^a^	0.246*	0.297*	0.189*	0.218*
Anxiety^a^	0.038	0.186*	0.054	0.068
Stress^a^	0.054	0.245*	0.175	0.144

*Note:* This table presents Pearson correlation coefficients showing the relationships between paired psychological variables (FCR, depression, anxiety, and stress) for survivors and their caregivers, highlighting interdependencies within the dyads.

Abbreviation: FCR, fear of recurrence.

* *p* < 0.05.

** *p* < 0.001.

^a^ Patients' variables.

^b^ Caregivers' variables.

Multivariable logistic regression analysis identified several independent variables associated with a high FCR in lymphoma patients. A time since diagnosis of less than 3 years (adjusted odds ratio [aOR]: 5.135, 95% CI: 1.852–14.238, *p* = 0.002), patients anxiety (aOR: 2.540, 95% CI: 1.014–6.363, *p* = 0.002), low FACT‐Lym score (aOR: 4.279, 95%CI: 1.738–10.531, *p* = 0.002) and high caregiver FCR (aOR: 2.970, 95% CI: 1.119–7.879, *p* = 0.029) were significantly associated with a high FCR (Table 5). In the univariable analyses, disease recurrence (OR: 3.122, 95%CI: 1.191–8.186, *p* = 0.021) and depression (OR: 2.095, 95%CI: 0.948–4.629, *p* = 0.067) were associated with high FCR, though these did not remain significant in the multivariable model.

**TABLE 5:** Univariable and multivariable‐adjusted logistic regression analyses of factors associated with high FCR in lymphoma patients.

	Univariable			Multivariable		
	OR	95% CI	*p*	OR	95% CI	*p*
Age (continuous)	0.978	0.953–1.004	0.092	**0.975**	0.944–1.008	0.143
Sex (female vs. male)	1.514	0.732–3.135	0.264			
Education (university or above vs. high school or below)	1.522	0.733–3.158	0.260			
Type of lymphoma (NHL vs. HL)	1.515	0.718–3.198	0.276			
Time since diagnosis (< 3 vs. ≥ 3)	3.214	1.475–7.004	0.003	5.135	1.852–14.238	0.002
Recurrence (yes vs. no)	3.122	1.191–8.186	0.021	3.049	0.932–9.973	0.065
Chemotherapy (yes vs. no)	1.304	0.515–3.305	0.575			
Depression (yes vs. no)	2.095	0.948–4.629	0.067	1.172	0.404–3.398	0.770
Anxiety (yes vs. no)	2.826	1.339–5.966	0.006	2.540	1.014–6.363	0.002
Stress (yes vs. no)	1.745	0.717–4.249	0.220			
FACT‐Lym (total) (low vs. high)	6.186	2.777–13.778	< 0.001	4.279	1.738–10.531	0.002
Caregiver's FCR	3.339	1.554–7.176	0.002	2.970	1.119–7.879	0.029
Caregiver's depression	1.796	0.851–3.791	0.124			
Caregiver's anxiety	1.724	0.829–3.584	0.145			
Caregiver's stress	1.731	0.764–3.921	0.189			

Abbreviations: CI, confidence interval; FACT‐Lym, the functional assessment of cancer therapy lymphoma; FCR, fear of recurrence; HL, Hodgkin lymphoma; NHL, non‐Hodgkin lymphoma; OR, odd ratio.

## 4 Discussion

Our study revealed that high FCR was prevalent among lymphoma survivors and their caregivers. Factors associated with high FCR in lymphoma survivors included poor overall QoL, recent lymphoma diagnosis, patient anxiety, and caregiver FCR. To our knowledge, this is the first study to simultaneously examine FCR, QOL, depression, anxiety, and stress levels in lymphoma survivors and their caregivers, as well as perform correlation analysis for paired psychological variables.

Our study found a lower prevalence (50.8%) of lymphoma survivors with high levels of FCR compared to a prior meta‐analysis (80%) that included leukemia and NHL survivors [10]. In contrast, a higher prevalence of high FCR was reported among French lymphoma patients (44%) [26]. A previous study in Germany among patients with hematological cancers found low levels of FCR (36%) [27]; however, the majority of participants had acute myeloid leukemia, making direct comparisons challenging. This discrepancy could potentially be attributed to the utilization of different FCR measurement tools (FoP‐Q‐SF vs. FCR‐SF), as research suggests these instruments can influence the reported prevalence [28]. Further, FCR is not confined to just patients, our data demonstrated that caregivers exhibited higher levels of FCR compared to the patients themselves. This finding aligns with previous research on caregivers of prostate cancer patients [29] and a recent study revealing elevated FCR in spouses compared to lymphoma survivors [30]. This is possible because the patients were hospitalized, and existing studies have shown that active treatment and ongoing medical evaluations can alleviate FCR in this population, conversely, caregivers experience these indirectly, potentially contributing to increased anxiety about recurrence [31].

Our study demonstrated a significant association between caregiver FCR and elevated FCR in lymphoma patients. Caregivers, including partners, parents, children, friends, or relatives, often take on the role of unpaid informal caregivers for cancer patients [32]. Several studies have shown that family caregivers may experience greater FCR than the survivors themselves, with patients' FCR closely linked to caregivers' FCR [15, 33]. However, variables related to the survivor‐caregiver relationship did not consistently predict caregivers' FCR. Qualitative data suggest fluctuations in caregivers' FCR, with heightened levels observed before follow‐up appointments, mirroring the experiences of survivors [34]. The interrelatedness of survivors' and caregivers' FCR and the persistent nature of FCR highlight the potential benefits of addressing FCR dyadically to achieve synergistic effects in couple‐based interventions, although such interventions may not be universally adopted [35]. Given that caregiver FCR is associated with psychological distress and decreased quality of life, which can compromise their caregiving capacity, addressing caregivers' FCR may lead to improved outcomes for both cancer survivors and their caregivers.

While previous research has consistently shown significant correlations between anxiety and FCR in various cancers, including head and neck, pancreatic, and testicular cancers [36, 37], findings specific to lymphoma survivors remain limited [38]. However, our results align with broader evidence demonstrating that anxiety disorder is independently associated with higher levels of FCR. Our findings support the hypothesis by van de Wal et al. [34] that FCR is a pervasive concern among cancer survivors, not limited to specific cancer types. The observed association between anxiety disorder and high FCR might be attributed to the correlation between insufficient or poor‐quality information and heightened anxiety. Anxiety has also been associated with impairments in cognitive domains, leading to difficulties in comprehending complex medical information and reduced patient satisfaction [39]. As a result, poorer mental health and emotional functioning have been associated with high FCR.

Lower overall QOL predicted higher FCR in our study. Existing data suggests a higher level of FCR is associated with poorer QOL in various cancer survivor populations, including colorectal, breast, periampullary, pancreatic, testicular, and genitourinary cancers [34, 40]. In a cross‐sectional study, Petzel et al. [40], identified that among survivors of pancreatic and periampullary tumors, anxiety disorders and low QOL were independently associated with clinically significant levels of FCR. Similarly, another study investigating breast cancer survivors reported that concerns regarding the impact of recurrence on one's role demonstrated the strongest association with reduced QoL, compared to anxieties about health, womanhood, and mortality [41]. Additionally, difficulties in these functional domains can trigger FCR. Walburg et al. [26] also found poorer OQL was significantly associated with clinically significant FCR in French lymphoma survivors.

In our study, although there was a trend, younger age, and female sex were not independently associated with high FCR. This finding is partially incongruent with extant literature, which has demonstrated a statistically significant association between younger age and FCR [42, 43]. It is noteworthy that systematic reviews have consistently identified female sex as the most robust sociodemographic variable linked to elevated FCR scores [10]. Also, a recent large multicenter study of 1195 advanced cancer patients revealed that younger age and female sex were significantly associated with high levels of FCR [5]. The lack of statistical significance in our findings may reflect the influence of sample‐specific characteristics or limited statistical power, even though our sample size is not small (*n* = 118). Future studies employing larger patient cohorts are warranted to confirm the relationship between FCR, age, and sex.

There are some limitations that need to be considered when interpreting our findings. First, the study included a relatively small number of patients, particularly for subgroup analysis for lymphoma subgroups. Studies with larger sample sizes are necessary to confirm these findings. Second, while we used standardized, validated questionnaires, we did not evaluate the quality of the caregiver‐survivor relationship, which could influence the connection between their FCR [44]. Third, we used a cut‐off score of 13 for FCRI‐SF as it is the most commonly utilized in the literature; however, alternative cut‐off scores, such as 16 and 22, are also available and could yield different results. Additionally, the survey was conducted in a single hospital, and although the study involved nationwide patients, this may limit the generalizability of the findings. Finally, chronic physical symptoms such as pain and fatigue, which are common among cancer survivors and may contribute to FCR, were not assessed in our study. However, despite these limitations, our study provides valuable insights into the psychological challenges faced by lymphoma patients and their caregivers.

## 5 Conclusions

Our study highlighted that approximately half of the lymphoma survivors (50.8%) and their caregivers (57.6%) reported high levels of FCR. Among lymphoma survivors, factors associated with high FCR included poor overall QoL, a diagnosis within the past 3 years, survivor anxiety, and caregiver FCR. Our findings demonstrate that survivor FCR is significantly associated with caregiver FCR but not with caregiver anxiety, suggesting that FCR may reflect specific concerns about cancer recurrence that are distinct from generalized anxiety. This divergence highlights the need for interventions tailored to address FCR directly, as traditional anxiety‐focused therapies may not fully address these fears in caregivers or survivors. These findings emphasize the necessity for comprehensive, multidimensional care strategies to effectively address FCR, thereby enhancing QOL and psychological well‐being. Implementing targeted interventions and support mechanisms for both patients and caregivers can significantly mitigate FCR and improve overall treatment outcomes.

## Author Contributions

**Taha Koray Sahin:** conceptualization, data curation, formal analysis, funding acquisition, investigation, methodology, project administration, resources, software, visualization, writing – original draft. **Ezgi Aysu Sahin:** data curation, formal analysis. **Hande Nur Gungor:** data curation, formal analysis. **Deniz Can Guven:** conceptualization, data curation, formal analysis, funding acquisition, investigation, methodology, project administration, resources, software, supervision, validation, visualization, writing – review and editing. **Ibrahim Barista:** conceptualization, data curation, formal analysis, funding acquisition, investigation, methodology, project administration, resources, software, supervision, validation, visualization, writing – review and editing. **Serkan Akin:** conceptualization, data curation, formal analysis, funding acquisition, investigation, methodology, project administration, resources, software, supervision, validation, visualization, writing – review and editing.

## Ethics Statement

This study was performed in line with the principles of the Declaration of Helsinki. Approval was granted by the Ethics Committee on Hacettepe University (IRB Ref No: SBA 24/457).

## Consent

All the participants provided written informed consent.

## Conflicts of Interest

The authors declare no conflicts of interest.

## Data Availability

The data that support the findings of this study are available from the corresponding author upon reasonable request.

## References

[1] E. Campo , E. S. Jaffe , J. R. Cook , et al., “The International Consensus Classification of Mature Lymphoid Neoplasms: A Report From the Clinical Advisory Committee,” Blood 140 (2022): 1229–1253, 10.1182/blood.2022015851. 35653592 PMC9479027

[2] S. Xie , Z. Yu , A. Feng , et al., “Analysis and Prediction of Relative Survival Trends in Patients With Non‐Hodgkin Lymphoma in the United States Using a Model‐Based Period Analysis Method,” Frontiers in Oncology 12 (2022): 942122, 10.3389/fonc.2022.942122. 36237337 PMC9551310

[3] B. Vannata , A. Conconi , J. Winkler , et al., “Late Relapse in Patients With Diffuse Large B‐Cell Lymphoma: Impact of Rituximab on Their Incidence and Outcome,” British Journal of Haematology 187 (2019): 478–487. 31385291 10.1111/bjh.16106

[4] A. C. Lo , A. Liu , Q. Liu , et al., “Late Cardiac Toxic Effects Associated With Treatment Protocols for Hodgkin Lymphoma in Children,” JAMA Network Open 7 (2024): e2351062, 10.1001/jamanetworkopen.2023.51062. 38241048 PMC10799264

[5] C. Calderon , M. Gustems , R. Galán‐Moral , M. M. Muñoz‐Sánchez , L. Ostios‐García , and P. Jiménez‐Fonseca , “Fear of Recurrence in Advanced Cancer Patients: Sociodemographic, Clinical, and Psychological Correlates,” Cancers 16 (2024): 909. 38473270 10.3390/cancers16050909 PMC10931223

[6] S. J. Kim , D. Kang , I. R. Kim , et al., “Impact of Fear of Cancer Recurrence on Survival Among Lymphoma Patients,” Psycho‐Oncology 29 (2020): 364–372, 10.1002/pon.5265. 31654534

[7] L. E. Latella , M. Rogers , H. Leventhal , et al., “Fear of Cancer Recurrence in Lymphoma Survivors: A Descriptive Study,” Journal of Psychosocial Oncology 38 (2020): 251–271, 10.1080/07347332.2019.1677840. 31617830 PMC7159999

[8] B. Thewes , P. Butow , R. Zachariae , S. Christensen , S. Simard , and C. Gotay , “Fear of Cancer Recurrence: A Systematic Literature Review of Self‐Report Measures,” Psychooncology 21 (2012): 571–587, 10.1002/pon.2070. 22021099

[9] A. B. Smith , D. Costa , J. Galica , et al., “Spotlight on the Fear of Cancer Recurrence Inventory (FCRI),” Psychology Research and Behavior Management 13 (2020): 1257–1268, 10.2147/prbm.S231577. 33376421 PMC7762428

[10] Y. L. Luigjes‐Huizer , N. M. Tauber , G. Humphris , et al., “What Is the Prevalence of Fear of Cancer Recurrence in Cancer Survivors and Patients? A Systematic Review and Individual Participant Data Meta‐Analysis,” Psycho‐Oncology 31 (2022): 879–892, 10.1002/pon.5921. 35388525 PMC9321869

[11] D. C. Güven , M. S. Y. Thong , and V. Arndt , “Survivorship Outcomes in Patients Treated With Immune Checkpoint Inhibitors: A Scoping Review,” Journal of Cancer Survivorship (2024), 10.1007/s11764-023-01507-w. PMC12081552 38175366

[12] K. D. Miller , L. Nogueira , T. Devasia , et al., “Cancer Treatment and Survivorship Statistics, 2022,” CA: a Cancer Journal for Clinicians 72 (2022): 409–436, 10.3322/caac.21731. 35736631

[13] E. M. Parry , S. Roulland , and J. Okosun , “DLBCL Arising From Indolent Lymphomas: How Are They Different?,” Seminars in Hematology 60 (2023): 277–284, 10.1053/j.seminhematol.2023.11.002. 38072721

[14] K. Webb , L. Sharpe , P. Butow , et al., “Caregiver Fear of Cancer Recurrence: A Systematic Review and Meta‐Analysis of Quantitative Studies,” Psychooncology 32 (2023): 1173–1191, 10.1002/pon.6176. 37303263

[15] A. Smith , V. S. Wu , S. Lambert , et al., “A Systematic Mixed Studies Review of Fear of Cancer Recurrence in Families and Caregivers of Adults Diagnosed With Cancer,” Journal of Cancer Survivorship 16 (2022): 1184–1219, 10.1007/s11764-021-01109-4. 34762248

[16] C. D. Bergerot , E. J. Philip , P. G. Bergerot , N. Siddiq , S. Tinianov , and M. Lustberg , “Fear of Cancer Recurrence or Progression: What Is It and What Can we Do About It?,” American Society of Clinical Oncology Educational Book 42 (2022): 1–10, 10.1200/edbk_100031. 35561298

[17] J. Lamarche , A. Cusson , R. Nissim , et al., “It's Time to Address Fear of Cancer Recurrence in Family Caregivers: Usability Study of an Virtual Version of the Family Caregiver‐Fear of Recurrence Therapy (FC‐FORT),” Frontiers in Digital Health 5 (2023): 1129536, 10.3389/fdgth.2023.1129536. 37671170 PMC10475944

[18] S. Mellon , T. S. Kershaw , L. L. Northouse , and L. Freeman‐Gibb , “A Family‐Based Model to Predict Fear of Recurrence for Cancer Survivors and Their Caregivers,” Psychooncology 16 (2007): 214–223, 10.1002/pon.1074. 16906624

[19] S. Simard and J. Savard , “Fear of Cancer Recurrence Inventory: Development and Initial Validation of a Multidimensional Measure of Fear of Cancer Recurrence,” Supportive Care in Cancer 17 (2009): 241–251. 18414902 10.1007/s00520-008-0444-y

[20] S. Simard and J. Savard , “Screening and Comorbidity of Clinical Levels of Fear of Cancer Recurrence,” Journal of Cancer Survivorship 9 (2015): 481–491, 10.1007/s11764-015-0424-4. 25603948

[21] L. Koral and Y. Cirak , “The Relationships Between Fear of Cancer Recurrence, Spiritual Well‐Being and Psychological Resilience in Non‐metastatic Breast Cancer Survivors During the COVID‐19 Outbreak,” Psycho‐Oncology 30 (2021): 1765–1772, 10.1002/pon.5727. 33982371 PMC8237000

[22] P. F. Lovibond and S. H. Lovibond , “The Structure of Negative Emotional States: Comparison of the Depression Anxiety Stress Scales (DASS) With the Beck Depression and Anxiety Inventories,” Behaviour Research and Therapy 33 (1995): 335–343, 10.1016/0005-7967(94)00075-u. 7726811

[23] Ö. Yılmaz , H. Boz , and A. Arslan , “Depresyon Anksiyete Stress Ölçeğinin (DASS21) Türkçe Kısa Formunun Geçerlilik‐Güvenilirlik Çalışması,” Finans Ekonomi Ve Sosyal Araştırmalar Dergisi 2 (2017): 78–91.

[24] F. J. Hlubocky , K. Webster , J. Cashy , J. Beaumont , and D. Cella , “The Development and Validation of a Measure of Health‐Related Quality of Life for Non‐Hodgkin's Lymphoma: The Functional Assessment of Cancer Therapy—Lymphoma (FACT‐Lym),” Lymphoma 2013 (2013): 147176, 10.1155/2013/147176.

[25] M. G. Sezgin and H. Bektaş , “Symptom Clustering and Its Effect on Functional Status in Lymphoma Patients,” Florence Nightingale Journal of Nursing 28 (2020): 143–154, 10.5152/fnjn.2020.19107. 34263193 PMC8152162

[26] V. Walburg , M. Rueter , S. Lamy , et al., “Fear of Cancer Recurrence in Non‐ and Hodgkin Lymphoma Survivors During Their First Three Years of Survivorship Among French Patients,” Psychology, Health & Medicine 24 (2019): 781–787, 10.1080/13548506.2019.1574354. 30714815

[27] S. Sarkar , A. Scherwath , L. Schirmer , et al., “Fear of Recurrence and Its Impact on Quality of Life in Patients With Hematological Cancers in the Course of Allogeneic Hematopoietic SCT,” Bone Marrow Transplantation 49 (2014): 1217–1222, 10.1038/bmt.2014.139. 25000458

[28] S. Simard , B. Thewes , G. Humphris , et al., “Fear of Cancer Recurrence in Adult Cancer Survivors: A Systematic Review of Quantitative Studies,” Journal of Cancer Survivorship 7 (2013): 300–322. 23475398 10.1007/s11764-013-0272-z

[29] L. M. Wu , H. McGinty , A. Amidi , K. Bovbjerg , and M. A. Diefenbach , “Longitudinal Dyadic Associations of Fear of Cancer Recurrence and the Impact of Treatment in Prostate Cancer Patients and Their Spouses,” Acta Oncologica 58 (2019): 708–714, 10.1080/0284186x.2018.1563714. 30741082 PMC6534441

[30] J. Wu , X. Lan , Z. Liao , J. Chen , Y. Wu , and R. Hu , “Comparison of the Sense of Spousal Support, Anxiety, Depression and Their Relationship to Fear of Cancer Recurrence Between Lymphoma Patients and Their Spouses: A Cross‐Sectional Study in China,” Journal of Cancer Survivorship (2023), 10.1007/s11764-023-01443-9. 37610477

[31] J. T. W. Williams , A. Pearce , and A. Smith , “A Systematic Review of Fear of Cancer Recurrence Related Healthcare Use and Intervention Cost‐Effectiveness,” Psychooncology 30 (2021): 1185–1195, 10.1002/pon.5673. 33880822

[32] V. Sun , D. J. Raz , and J. Y. Kim , “Caring for the Informal Cancer Caregiver,” Current Opinion in Supportive and Palliative Care 13 (2019): 238–242. 31157656 10.1097/SPC.0000000000000438 PMC6669089

[33] A. A. Cohee , R. N. Adams , S. A. Johns , et al., “Long‐Term Fear of Recurrence in Young Breast Cancer Survivors and Partners,” Psycho‐Oncology 26 (2017): 22–28. 26490953 10.1002/pon.4008 PMC4840075

[34] M. van de Wal , L. van de Poll‐Franse , J. Prins , and M. Gielissen , “Does Fear of Cancer Recurrence Differ Between Cancer Types? A Study From the Population‐Based PROFILES Registry,” Psychooncology 25 (2016): 772–778, 10.1002/pon.4002. 26464337

[35] H. Badr , J. Bakhshaie , and K. Chhabria , “Dyadic Interventions for Cancer Survivors and Caregivers: State of the Science and New Directions,” Seminars in Oncology Nursing 35 (2019): 337–341, 10.1016/j.soncn.2019.06.004. 31248677 PMC6660372

[36] N. Ghazali , E. Cadwallader , D. Lowe , G. Humphris , G. Ozakinci , and S. N. Rogers , “Fear of Recurrence Among Head and Neck Cancer Survivors: Longitudinal Trends,” Psychooncology 22 (2013): 807–813, 10.1002/pon.3069. 22451036

[37] T. Skaali , S. D. Fosså , R. Bremnes , et al., “Fear of Recurrence in Long‐Term Testicular Cancer Survivors,” Psychooncology 18 (2009): 580–588, 10.1002/pon.1437. 18855944

[38] C. Borreani , S. Alfieri , L. Farina , E. Bianchi , and P. Corradini , “Fear of Cancer Recurrence in Haematological Cancer Patients: Exploring Socio‐Demographic, Psychological, Existential and Disease‐Related Factors,” Support Care Cancer 28 (2020): 5973–5982, 10.1007/s00520-020-05434-9. 32285261

[39] O. Husson , F. Mols , and L. V. van de Poll‐Franse , “The Relation Between Information Provision and Health‐Related Quality of Life, Anxiety and Depression Among Cancer Survivors: A Systematic Review,” Annals of Oncology 22 (2011): 761–772, 10.1093/annonc/mdq413. 20870912 PMC3065875

[40] M. Q. Petzel , N. H. Parker , A. D. Valentine , et al., “Fear of Cancer Recurrence After Curative Pancreatectomy: A Cross‐Sectional Study in Survivors of Pancreatic and Periampullary Tumors,” Annals of Surgical Oncology 19 (2012): 4078–4084, 10.1245/s10434-012-2566-1. 22875648 PMC11976899

[41] L. E. Simonelli , S. D. Siegel , and N. M. Duffy , “Fear of Cancer Recurrence: A Theoretical Review and Its Relevance for Clinical Presentation and Management,” Psychooncology 26 (2017): 1444–1454, 10.1002/pon.4168. 27246348

[42] A. B. Kornblith , M. Powell , M. M. Regan , et al., “Long‐Term Psychosocial Adjustment of Older vs Younger Survivors of Breast and Endometrial Cancer,” Psychooncology 16 (2007): 895–903, 10.1002/pon.1146. 17245695

[43] D. L. Hall , L. I. Wagner , S. Lebel , A. B. Smith , C. D. Bergerot , and E. R. Park , “Guidelines Needed for the Management of Fear of Cancer Recurrence in Adult Survivors of Cancer in the United States: A Consensus Statement,” Cancer 130 (2024): 2739–2742, 10.1002/cncr.35326. 38630904 PMC11286349

[44] R. Maguire , P. Hanly , M. Balfe , et al., “Worry in Head and Neck Cancer Caregivers: The Role of Survivor Factors, Care‐Related Stressors, and Loneliness in Predicting Fear of Recurrence,” Nursing Research 66 (2017): 295–303. 28654567 10.1097/NNR.0000000000000223

